# Adapting the EQ-5D-3L for adults with mild to moderate learning disabilities

**DOI:** 10.1186/s12955-024-02254-x

**Published:** 2024-04-29

**Authors:** John L. O’Dwyer, Louise D. Bryant, Claire Hulme, Paul Kind, David M. Meads

**Affiliations:** 1 Academic Unit of Health Economics, Leeds Institute of Health Sciences, University of Leeds, Worsley Building, Leeds, LS2 9NL UK; 2 Division of Psychological & Social Medicine, Leeds Institute of Health Sciences, School of Medicine, University of Leeds, Worsley Building, Leeds, LS2 9JT UK; 3 Department of Health & Community Sciences, University of Exeter Medical School, South Cloisters, St Luke’s Campus, Exeter, EX1 2LU UK; 4 Department of Applied Health Research, University College London, Gower Street, London, WC1E 6BT UK

**Keywords:** EQ-5D, Learning disability, Intellectual disability, Health-related quality of life, Patient-reported outcome measures.

## Abstract

**Background:**

Approximately 1.5 million adults in the UK have a learning disability. The difference between age at death for this group and the general population is 26 years for females and 22 years for males. The NHS Long Term Plan (January 2019) recognises learning disabilities as a clinical priority area. People with a learning disability are often excluded from research by design or lack of reasonable adjustments, and self-reported health status/health-related quality of life questionnaires such as the EQ-5D are often not appropriate for this population. Here, we systematically examine the EQ-5D-3L (its wording, content, and format) using qualitative methods to inform the adaption of the measure for use with adults with mild to moderate learning disabilities.

**Methods:**

Think-aloud interviews with carers/advocates of learning-disabled adults were undertaken to explore the difficulties with completing the EQ-5D-3L. Alternative wording, language, structure, and images were developed using focus groups, stakeholder reference groups, and an expert panel. Data analysis followed a framework method.

**Results:**

The dimensions and levels within the EQ-5D-3L were deemed appropriate for adults with mild to moderate learning disabilities. Consensus on wording, structure, and images was reached through an iterative process, and an adapted version of the EQ-5D-3L was finalised.

**Conclusion:**

The EQ-5D-3L adapted for adults with mild to moderate intellectual/learning disabilities can facilitate measurement of self-reported health status. Research is underway to assess the potential use of the adaptation for economic evaluation.

**Supplementary Information:**

The online version contains supplementary material available at 10.1186/s12955-024-02254-x.

## Introduction

There are approximately 1.5 million people with a learning disability in the United Kingdom, most of whom have mild to moderate learning disabilities [1]. These individuals experience significant health inequalities and have significantly higher rates of mortality and morbidity than people without a learning disability [2]. In 2021, males and females died 22 and 26 years younger than the average population [3].

The January 2019 NHS Long Term Plan recognised learning disabilities as a clinical priority area [4]. However, people with learning disabilities are often excluded from research addressing health inequalities due to assumptions about mental capacity or lack of reasonable adjustments [5]. This includes cost-effectiveness research to assess value for money. Data are needed for economic evaluations; collecting both cost and health-related quality of life (HRQoL) data. There are valid concerns about the reliability of gathering resource use data from people with learning disabilities due to challenges in completion, specifically around recall; however, electronic records can often fill this gap [6]. The difficulties researchers face with collecting HRQoL data for this population are not so easily resolved. A recent systematic review of literature on how the effects of interventions for people with learning disabilities could be measured in economic evaluations suggested using techniques tailored to people with learning disabilities, such as specifically developed preference-based instruments [7].

NICE guidance recommends that changes in HRQoL should be patient- or proxy-reported, relying on proxies when self-reporting is not feasible [8]. The validity of using proxy reporting has been explored in several studies, finding that proxies often report more health limitations and may not be accurate in some aspects [9–11]. NICE’s preferred measure to generate quality-adjusted life years (QALYs) is the EQ-5D, a standardised generic instrument available as a proxy version [8]. The EQ-5D has five domains (mobility, self-care, usual activities, pain/discomfort, and anxiety/depression). In the three-level version, the EQ-5D-3L, each domain has three response options which indicate no problems to extreme problems in relation to ‘*your own health today’. *A five-level (EQ-5D-5L) version has additional in-between levels, providing richer data [12].

A recent study indicated that the EQ-5D-3L should not be used in its current form in adults with learning disabilities as, although rephrasing and explanations facilitated completion, a substantial proportion of participants had difficulty understanding the questions [6]. It has been suggested that it would be unrealistic to expect that changing the wording alone would deliver an appropriate measure; supporter or researcher involvement will almost always be required [13]. The onus is on researchers to provide materials that enable adults with learning disabilities the opportunity to self-report, with support if required, on their own health status or quality of life. Whilst there has been debate regarding the use of QALYs within this population, our work aligns with recommendations for future economic evaluations, specifically addressing limitations of the original EQ-5D-3L [7]. This paper reports on an approach using qualitative methods to develop a reliable and standardised method of collecting self-reported HRQoL data, namely by adapting the EQ-5D-3L for adults with mild to moderate learning disabilities.

## Methods

A systematic review of existing research which identified developments and adaptations of self-reported QoL/HRQoL research measures in this population was completed by the authors. This study was registered on PROSPERO (CRD42018092423, 2018) (see Appendix 1 for a list of adapted measures). Frequently the response rate for measures or the success of adaptations to existing measures were not reported. Levels of missing data were rarely reported in the studies, therefore it was not possible to make recommendations on the feasibility of these measures. However, common adaptations used such as pictograms, paraphrasing, and longer completion times served as a reference for this development phase.

### Development of the adapted EQ-5D

Throughout the development phase, the principal researcher consulted with *easy on the i*, an information design service specialising in creating easy-read health information within the Learning Disability Service at Leeds and York Partnership NHS Foundation Trust. This service works closely with reference groups of adults with learning disabilities, who play a critical role in the development and design of materials. Groups are presented with images, symbols, photographs, and graphics, and are asked to share their interpretations and feedback on what these convey. Conversely, they are given concepts or ideas and asked to suggest appropriate visual representations. This iterative process ensures that materials are accessible and meaningful to adults with a learning disability, thereby enhancing the effectiveness of easy-read health information [14].

Development took place over five stages: (1) in-person think-aloud interviews with carers and supporters of adults with learning disabilities; (2) two in-person focus groups with interviewees; (3) expert advisory panel input; (4) iterative design and discussion with adults with learning disabilities; (5) final co-author consensus.

1. In-person Think Aloud interviews were conducted with carers and supporters of adults with learning disabilities recruited through networks in the West Yorkshire region. In cognitive interviewing, the think-aloud technique requires participants to articulate their thought process aloud while engaging in a task and facilitates an understanding of their cognitive reasoning for the interviewer. This interview style enables an in-depth exploration of how responders complete patient-reported outcome measures [15–17]. Interviewees were asked to choose between the EQ-5D versions (-3L or -5L) and to imagine that they were completing it with someone with a learning disability. They were guided to think aloud to clarify complex words or concepts they judged their person would have difficulty understanding. The process started with initial EQ-5D instructions and proceeded through each domain, followed by a semi-structured interview and verbal probing of each element of the EQ-5D. Interviewees were asked to relate each domain to the life of the person they support and assess its relevance. They were prompted to discuss the wording, content, format, and the domains or levels included or potentially missing from the measure.
2. Interview findings were presented to two focus groups of carers and supporters who had participated in the qualitative interviews. A topic guide, similar to the interview structure, facilitated discussion on adapting the EQ-5D’s layout, domains, and levels. Preliminary wording and initial images from the *easy on the i *image bank were also discussed [14].
3. Development stage results were presented to an advisory panel of stakeholders and academics specialising in the area of quality of life measurement, learning disability or information design. This panel included a diverse group of members such as university academics; a learning disability information design service manager; clinical leads for learning disability in Leeds Teaching Hospitals Trust and Leeds Clinical Commissioning Group; and the co-ordinator for learning disability advocacy services in Leeds (Full list in Acknowledgements). Findings from these first three stages were reviewed and incorporated.
4. An iterative discussion and design process began with *easy on the i* reference groups and their in-house designer to refine the proposed questionnaire. Changes were consolidated through input from a focus group of participants with learning disabilities from a third-sector advocacy group Connect In The North.
  The World Health Organisation’s classification of the severity of learning disabilities uses IQ to determine if a person is mildly, moderately, severely, or profoundly disabled. This study adopted an inclusive recruitment approach; participants with learning disabilities did not undergo IQ tests, as this would potentially add to the exclusion of this group from research. Participants’ comprehension and communication abilities varied. Prior to recruitment, an advocate/supporter, acting as a gatekeeper, informed adults with learning disabilities, whom they discerned had the ability to take part, about the study, and facilitated researcher access if the potential participant was interested. All recruited participants had the capacity to consent.

(5) The authors reached a consensus on the final version of the adapted EQ-5D-3L.
  All interviews and focus groups were audio-recorded and transcribed to ensure accuracy in data representation. Data were analysed using NVivo software (QSR International Pty Ltd. Version 12, 2018). Analysis of the data followed the Framework Method developed by Ritchie and Spencer [18]. This method is commonly used for the thematic analysis of semi-structured interview transcripts where the question domains are predefined, facilitating categorisation, in this case, around the original EQ-5D domains. The process followed several stages. Firstly authors JOD and LB familiarised themselves with the data and agreed upon the categories. Codes capturing key concepts and insights were grouped into categories aligned with the original EQ-5D domains. The categorised data were then systematically charted across the full dataset to compare responses and to ensure full coverage of participants’ experiences. Finally, the charted data was combined, and patterns were identified that adhered to the original EQ-5D framework and highlighted other relevant findings.
  By following the Framework Method, we ensured that the analysis was thorough and methodical while accommodating fresh insights.

## Results

### Stage 1: think aloud interviews

Between April and July 2019, 14 interviews were completed with carers and paid supporters (12 females, two males) of adults with learning disabilities. Six participants classified themselves as carers, six as paid supporters, and two were carers who also worked as paid supporters of adults with learning disabilities. Participant ages ranged from 32 to 88 (mean 57 years). Interviews took pace at a location of the participants choosing; either at their home, their place of work or at the University of Leeds. Data saturation was apparent by the twelfth interview, but due to a gender imbalance, two additional male participants were interviewed. Interviews lasted 60–90 min.

All participants favoured using the EQ-5D-3L over the EQ-5D-5L, as it was clearer and potentially less overwhelming for someone with a learning disability. Initial comments focused on the layout of the measure first; the need for larger text in an easy-read format was the predominant initial reaction. Ten of 14 participants recommended incorporating images or photographs, and four suggested using different text and paper colours.

Five participants advised simplifying the opening statement and repeating the word ‘today’ within each domain to focus responses on the current day and not a time in the past.

*“…she’d have forgotten about that. I’d have had to keep going back to that and saying, ‘No, today! Not what you do on a Sunday with your PA’ you know?” – P9*.

*“I’d have to do that with my daughter for example, because she would say, ‘Well do you remember when I had really bad toothache Mum, and you had to take me to dentist?’; [Laughs] ‘Well I do, but we’re talking about today!’. ‘And also, do you remember when I hurt my arm?’; ‘Yes I know, but we’re talking about today!’ – P6*.

Regarding the wording of the levels, all 14 interviewees suggested replacing ‘moderate’ with ‘a bit’ or ‘some,’ and ‘extreme’ with ‘a lot’. Three interviewees questioned the use of the word ‘problem’, suggesting it has negative connotations, advocating for the more neutral ‘difficulty’.

*“Don’t know about the word problem? Or whether that should be difficulty rather than any problem? And whether that’s … could be taken a bit negatively. I’m not sure about that.” – P11*.

*“It’s coming into my head like, ‘Erm, have you had problems? Have you had problems walking about?’ but why put it as a problem? What about a person who is in a wheelchair who has been in a wheelchair all their life? It’s not a problem to them, that’s how they live.” – P8*.

Regarding the Mobility domain, 12 interviewees suggested that ‘Mobility’ meant ‘Getting about’ or ‘Moving around’. While four interviewees thought ‘Mobility’ was clear, three among them believed it implied more than walking. All acknowledged that the levels specifically referred to the ability to walk:

*“How do you get about? Yes… because it’s not just walking…if you were in a wheelchair, how do you get on the bus? How do you get in a taxi? That’s asking just about walking.” – P3*.

*“….somebody with a wheelchair might not have any issues with mobility because they get in their own wheelchair! What is it that you’re wanting to know about people?…[Laughs]” – P12*.

All interviewees felt that the most severe mobility level, ‘confined to bed’, should be rephrased:

*“We have had people with learning disabilities saying that they don’t want to go to bed early. They’re made to go to bed early so the carers can go home! And they don’t want to go to bed at six o’clock at night or eight o’clock at night. So will they think that is ‘I’ve been confined to my bedroom’?” – P5*.

*“Your first two questions are clearly about walking, and I wouldn’t ask if people were confined to bed…so yeah, if it’s about walking, then that question needs to be ‘I cannot walk’ – P12*.

Most interviewees (12/14) stated that they would change the words ‘Self-Care’ to ‘Looking after yourself’:

*“I have no problems with self-care…well that’s just looking after yourself”-P2*.

*“I’ve no problems with self-care…erm…I’d be putting washing and dressing or looking after yourself in brackets. I have some problems washing or dressing myself…I am unable to wash or dress myself…Yes they’re fine. It’s just self-care needs changing.” – P3*.

When considering ‘washing and dressing’, all interviewees stated that ‘Self-Care’ covered more than just these activities. Examples of activities they included as self-care were brushing teeth, brushing and drying hair, eating, shaving, taking medication, cooking, and personal hygiene. Two interviewees advised that asking about multiple self-care activities could overwhelm or confuse a person with a learning disability, suggesting that ‘washing and dressing’ was sufficient.

The term ‘Usual activities’ raised questions for several interviewees. They felt that some of the examples listed in the EQ-5D might not be appropriate for adults with learning disabilities.

*“What does usual mean…by whose standard you know? Somebody who is learning disabled or somebody who’s not?” – P5*.

*“What usual activities? So, obviously she doesn’t work….Housework…She doesn’t…She will wash a few pots up…Erm… Family, she’d probably go like, ‘What do you mean? What activities do you mean?’ It’s a bit vague isn’t it, when it says family?” – P9*.

Interviewees suggested that people in this population often take statements literally. Therefore, it may be more effective to ask about their planned activities for the day and whether they could not perform these due to external factors like carer availability. This would account for cases where a lack of support disrupts usual activities.

*“It’s your health not your circumstance. So health might prevent somebody from going swimming. Well, I know of some that can’t get support workers to take them swimming, so and that’s the thing you really have to keep bringing them back on to. It’s your physical well-being, not that you’ve not got a support worker that’ll go swimming” – P5*.

*“Sometimes it’s not easy for people to do things because somebody else is stopping them rather than anything intrinsic to them…. But yeah, sometimes it’s not the person, it’s the support around them…And if there’s only one staff on and there’s four of you living in the house, either everybody goes out together or nobody goes out.” – P12*.

Most interviewees saw no issue in asking adults with learning disabilities about pain. However, four interviewees suggested that these individuals might not communicate their pain as quickly as those without learning disabilities.

*“They do find it quite difficult, some people, describing that and knowing that, because I suppose for us, like if you pull a muscle or something, you would know what you’d done… I suppose their awareness of their body is, it would be different, might be different because everybody has different pain thresholds.” – P11*.

*“Most people know what pain is, but my daughter has a high pain threshold, so we never know when she’s had an abscess. She can have like four abscesses and not tell you she’s in pain…she finds it difficult… She can be really poorly and not tell you” – P3*.

While most interviewees foresaw few issues with asking about pain, they suggested that “discomfort” could be removed from the questionnaire.

*“Yeah, well, I’m wondering whether I’d just leave it at pain? Whether I think discomfort is…is muddying the water? Discomfort is just being a bit uncomfortable, which isn’t the same as pain at all?”* – P12.

All interviewees rephrased the Anxiety/Depression domain. The language used to rephrase was common to all interviewees. ‘Anxious’ was rephrased as ‘worried’ by all interviewees. Other words used were: ‘Stressed’, ‘upset’, ‘frightened’, ‘nervous’, and ‘unsafe’.

‘Depressed’ was rephrased as ‘sad’ or ‘unhappy’ by all interviewees and ‘unhappy all the time’ by three. It was suggested that this domain could be introduced conversationally while asking the person about their mood today. Two interviewees suggested anxiety is inherent to living with a learning disability. More than half of the interviewees felt the questionnaire lacked a holistic outlook on quality of life, identifying a link between the feelings of anxiety or depression associated with loneliness and the lack of social opportunities or friendships that people with learning disabilities experience.

Despite being highly critical of the wording and layout in the EQ-5D-3L, no interviewee felt strongly that additional domains were needed if measuring health status was the objective. However, regarding the broader concept of quality of life, they thought it lacked vital elements of a well-being domain, missing out on “*the person*”. Three interviewees suggested that the EQ-5D-3L focused too much on physical health, emphasising that it is “*too clinical*” in its wording and domains.

All agreed that the current EQ-VAS scale was not suitable for adults with learning disabilities. Six interviewees suggested a horizontal scale from 0 to 10, left to right, with ‘0’ being ‘the worst’ you could feel and ‘10’ being ‘the best’. Removing the word ‘imagine’ was recommended to avoid confusion. Using pictograms (“*something like an emoji*”) with a sad and ‘smiley face’ at either end of the scale was suggested by interviewees as these would be familiar and understood by many people with a learning disability.

Interviewees found it challenging to think aloud and to simulate completing the measure for someone with a learning disability. They often vocalised that another person might have difficulty with other elements of the EQ-5D-3L, frequently highlighting the diverse abilities of the target population.

Interviewees considered communication to be the key factor for completing the EQ-5D-3L with someone with a learning disability; building a rapport with the person and being specific about the question would facilitate more accurate completion.

### Stage 2: focus groups

In September 2019, the interview findings were presented to the Think Aloud interviewees in two focus groups (*n* = 4; *n* = 5) to validate the interpretation and reach a consensus. Both in-person focus groups took place at the University of Leeds. A first iteration of an adapted EQ-5D-3L, which included the paraphrasing commonly suggested in the interviews and relevant images from the *easy on the i*image bank, was presented [14]. The new wording and images were discussed by focus group participants, who suggested minor changes they deemed necessary. One change that prompted much debate involved the Mobility and Self-Care domains, within which results from the interview data suggested replacing ‘*problems*’ with ‘*difficulty*’ within these categories. Following discussions, the focus group participants agreed that once adults with learning disabilities had the appropriate supports in place, they may not view themselves as having difficulty in terms of these domains; however, they would recognise the need for assistance, hence the changes to ‘*need help*’ in the mobility domain, and ‘*need some help*’ or ‘*need a lot of help from someone else*’ rather than ‘unable to’ in the Self-Care domain.

### Stage 3: advisory panel

In November 2019, a workshop with members (*n* = 12) of the project advisory panel consisting of stakeholders and academics was held. Panel members brought a range of expertise, from health economics and clinical practice to service user involvement and learning disability research. The diverse panel ensured a comprehensive review of the qualitative analysis and an updated iteration of the adapted EQ-5D. Their feedback affirmed the content validity of the adapted EQ-5D-3L questionnaire, suggesting minor changes to the format, wording, and images.

### Stage 4: adults who have learning disabilities input to the design

In July 2020, an updated version of an adapted EQ-5D-3L was presented to groups of adults with learning disabilities. The COVID-19 pandemic presented challenges, including the inability to meet research participants in person. The first meeting took place online via Zoom with an *easy on the i* reference group consisting of adults with learning disabilities and an accessible information designer. The meeting was facilitated by a service user involvement facilitator who was physically present with the reference group. Paper copies of all easy-read materials were provided in advance, and the adapted EQ-5D-3L was also shared on-screen. The group discussed each element of the adaptation, including the meaning and suitability of wording and images, and expressed their ideas on colours and formatting. Minor adjustments were made to the images, e.g., the position of the walking frame changed as the group thought it should be closer to the person in the image.

The next iteration of the adapted EQ-5D-3L was presented to an online focus group of six adults with learning disabilities from a centre for inclusive living in Leeds. All participants could read and were familiar with using Zoom regularly. This focus group followed a similar format to the previous meeting. Participants suggested changing the background to beige/yellow as “*it makes the white boxes easier for dyslexic people*”.

In August 2020, a final iteration was presented to a different *easy on the i* reference group (four adults) in a similar manner. No significant changes were required following this final consultation.

### Stage 5: final consensus meeting

Agreement on the finalised revisions to the proposed adaptation was reached among all contributing authors. This consensus encompassed approval of prior modifications and included refining the query from ‘what number shows how you are today?’ to ‘which number shows how you are today?‘.

## Discussion

As many people with learning disabilities have difficulty reading or understanding the original EQ-5D, the adapted wording was primarily informed by interviews and discussions with carers and supporters as they are the people who often relay concepts and terminology to the individuals they support. While this ensured a practical perspective, more input from people with learning disabilities on the wording may have yielded additional insights. There are challenges to identifying a representative group of people with learning disabilities who are also supported sufficiently to contribute to research. This study adopted an inclusive recruitment approach, deliberately avoiding IQ testing to prevent further exclusion; this follows a similar approach to previous research with this population [19]. Consequently, the participants with learning disabilities who assisted in the development of the adapted EQ-5D-3L may not have captured the entire spectrum of learning disabilities. Content validity was established through consensus with an advisory group, reflecting a comprehensive approach used throughout the wider study which included voices from both caregivers and the learning-disabled community.

The COVID-19 pandemic required changes in the method of engagement with participants, moving from in-person to online methods; this may have affected the depth and quality of feedback on the adapted measure. However, the presence of in-person facilitators and the comfortable, familiar settings for participants likely mitigated these effects. These limiting factors reflect a pragmatic approach to research under constrained circumstances. Despite the limitations created by the COVID-19 pandemic, the study achieved the objective of developing an adapted version of the EQ-5D-3L for adults with mild to moderate learning disabilities (see Appendix 4 - Reproduced by permission of EuroQol Research Foundation).

In the adapted version, the titles of three domains align with the EQ-5D-Y (the youth version). Notably, ‘Pain’ omits ‘Discomfort’; ‘Usual Activities’ is rephrased to ‘Doing things I want to do - because of how I am TODAY…’ to eliminate external factors which might cause difficulty.

Levels within domains remain similar, but with nuanced wording changes: ‘needing help’ replaces ‘having problems’ in Mobility and Self-Care, ‘difficulty’ supersedes ‘problems’ in Usual Activities, and ‘I am feeling OK’ replaces ‘I am not anxious or depressed’.

Carers of adults with learning disabilities confirmed that domains used in the EQ-5D-3L were transferable to this population and suggested the wording that should be used. An expert advisory panel and the authors discerned that these changes would improve comprehension and completion.

Previous attempts have been made to make the EQ-5D more accessible using images. Pictorial versions of EQ-5D were developed for people with aphasia. These versions used original EQ-5D wording together with line drawings which required additional development [20]. ﻿The images developed for our study were designed by an accessible information designer from an NHS Learning Disability Service and, combined with the wording of the adaptation, have been reviewed by reference groups of adults with learning disabilities.

Guidance for interviewers has been drafted based on findings from the earlier systematic review and the qualitative development stages. It includes instructions on what is permissible when supporting people with a learning disability to complete the measure. Responses can be verbal, marked with a pen, or pointed to. A supporter is welcome to observe or assist at the respondent’s discretion; however, they must not prompt or influence their answer. Only the respondent should provide answers, as this is not intended as a proxy measure. Frequent reminders to the respondent that they are being asked about ‘today’﻿ may be necessary.

Demonstrating the suitability of this adapted EQ-5D-3L for use by the target population is the next step towards using this as an outcome measure in economic evaluations in research with adults with learning disabilities. The authors have collected data on the adaptation’s performance (reliability and validity) compared to the original with the target population (manuscript in preparation). Given the apparent differences, assessing the extent to which valuations of health states using an adapted EQ-5D-3L correspond to the previously established measure when the same health states are valued is necessary. This research is underway.

## Conclusion

The aim of this paper is to report on the development of an adapted EQ-5D-3L for adults with mild to moderate learning disabilities, suitable for self-completion or with support if required. Adults with learning disabilities and their carers/supporters have assisted in the design. The adaptation will facilitate measuring health status and HRQoL in adults with a learning disability and support their inclusion in research.

### Supplementary Information

**Supplementary Material 1.**

**Supplementary Material 2.**

**Supplementary Material 3.**

**Supplementary Material 4.**

## Data Availability

Data are available from the corresponding author upon reasonable request. All Intellectual Property Rights in connection with the modified EQ-5D-3L for adults with intellectual/learning disabilities are vested in EuroQol. Reproduction of this version is not allowed. For reproduction, use or modification of the EQ-5D (any version), please register your study by using the online EQ registration page: www.euroqol.org.

## Abbreviations

EQ-5D: EuroQol Five Dimensions
EQ-5D-3L: EuroQol Five Dimensions – Three level
EQ-5D-5L: EuroQol Five Dimensions – Five level
EQ-VAS: EuroQol Visual Analogue Scale
HRQoL: Health-Related Quality of Life
IQ: Intelligence Quotient
NHS: National Health Service
NICE: National Institute for Health and Care Excellence
NVivo: NVivo software (QSR International Pty Ltd.)
QALY: Quality-Adjusted Life Years
QoL: Quality of Life
WHO: World Health Organisation
