# Supplementary material for: Adapting the EQ-5D-3L for adults with mild to moderate learning disabilities
Source: Health Qual Life Outcomes. 2024 Apr 29;22:37. doi: 10.1186/s12955-024-02254-x (PMC11059748; doi:10.1186/s12955-024-02254-x)
Supplement: Supplementary file 1 — Supplementary Material 1. [file 12955_2024_2254_MOESM1_ESM.pdf]

Appendix 1:

Adaptations and Innovations in Quality of Life Measures for Adults with Learning Disabilities

**Name of Measure**	**Adaptations/Innovations**
INTEGRAL Quality of Life Scale (1,2)	Recommended to be interview-administered for full comprehension
Life Satisfaction Scale (LSS) (4)	Advises that a proxy/supporter can be used for participants who are unable to communicate sufficiently well.<br>Advises taking extra time and sensitivity to build rapport with the participant
QOL-Q (5-21)	Instructions for participants to take their time, interviewers can mark the scale; allows rephrasing if the participant does not understand the original question and also allows a proxy to assist.
ICAC, Self-Concept Clinical Inventory (5);<br>Nottingham Health Profile (22);<br>QOL-Q (5-21);<br>SWLS (23,24)	Attempts to increase response rates by paraphrasing; changing wording to something more familiar/acceptable, using examples to elicit responses. Some, like QOL-Q, allow rephrasing.
HRQOL-IDD (25);<br>IDQOL (26,27);<br>LSS (4);<br>Mans-LD (28);<br>Personal Outcomes Scale (34-41);<br>PWI-ID (29-33);<br>SWLS (23,34)	Use of pictograms and/or images to aid self-reporting; e.g., "smiley faces" for simple emotional expressions, pictures accompanying statements on Mans-LD, and images of cups filled to various levels to represent a Likert scale on HRQOL-IDD.
IDQOL-16 (26,27)	Participants are advised to complete the measure by themselves but can be assisted by a supporter if needed.
Satisfaction with Life Scale (SWLS) (23,24).	Allows the researcher to read items out loud to the participant, who responds by saying the answer or by pointing to the applicable happy, neutral, or sad face. Time allowance for rephrasing and probing.
Life Circumstances Questionnaire (LCQ) (3), Personal Outcomes Scale (POS) (34-41)	Use an open-ended conversational interview as the method of completion. Eliciting examples and using "smiley faces" aids in understanding the POS items; a caregiver may also be asked to assist on the participant’s behalf.

References:

1. Badia, M., et al., *Relationships between leisure participation and quality of life of people with developmental disabilities.* Journal of applied research in intellectual disabilities : JARID, 2013. **26**(6): p. 533-45.

2. Gomez, L.E., et al., *Application of the Rasch rating scale model to the assessment of quality of life of persons with intellectual disability.* Journal of intellectual & developmental disability, 2012. **37**(2): p. 141-50.
3. Young, L. *Community and cluster centre residential services for adults with intellectual disability: long-term results from an Australian-matched sample*. Journal of intellectual disability research, 2006. **50**, 419-431

4. Emerson, E. and C. Hatton, *Self-reported well-being of women and men with intellectual disabilities in England.* American journal of mental retardation : AJMR, 2008. **113**(2): p. 143-55.
5. Albuquerque, C.P., *Psychometric properties of the Portuguese version of the Quality of Life Questionnaire (QOL-Q).* Journal of applied research in intellectual disabilities : JARID, 2012. **25**(5): p. 445-54.
6. Harner, C.J. and L.W. Heal, *The Multifaceted Lifestyle Satisfaction Scale (MLSS): psychometric properties of an interview schedule for assessing personal satisfaction of adults with limited intelligence.* Research in developmental disabilities, 1993. **14**(3): p. 221-36.
7. Caballo, C., et al., *Factor structure of the Schalock and Keith Quality of Life Questionnaire (QOL-Q): validation on Mexican and Spanish samples.* Journal of intellectual disability research : JIDR, 2005. **49**(Pt 10): p. 773-6.
8. Chou, Y.C., et al., *Outcomes of a new residential scheme for adults with intellectual disabilities in Taiwan: a 2-year follow-up.* Journal of intellectual disability research : JIDR, 2011. **55**(9): p. 823-31.
9. Tyrer, P., et al., *Neuroleptics in the treatment of aggressive challenging behaviour for people with intellectual disabilities: a randomised controlled trial (NACHBID).* Health Technology Assessment, 2009. **13**(37): p. 1-76.
10. Horovitz, M., et al., *The relationship between Axis I psychopathology and quality of life in adults with mild to moderate intellectual disability.* Research in developmental disabilities, 2014. **35**(1): p. 137-43.
11. Wong, P.K.S., et al., *Initial validation of the Chinese Quality of Life Questionnaire-Intellectual Disabilities (CQOL-ID): a cultural perspective.* Journal of intellectual disability research : JIDR, 2011. **55**(6): p. 572-80.

12. Lunsky, Y. and B.A. Benson, *Association between perceived social support and strain, and positive and negative outcome for adults with mild intellectual disability.* Journal of intellectual disability research : JIDR, 2001. **45**(Pt 2): p. 106-14.
13. Martin, G., et al. *An exploratory study of assertive community treatment for people with intellectual disability and psychiatric disorders: conceptual, clinical, and service issues*. Journal of intellectual disability research, 2005. **49**, 516-524 DOI: 10.1111/j.1365-2788.2005.00709.x.
14. Duvdevany, I. and E. Arar, *Leisure activities, friendships, and quality of life of persons with intellectual disability: foster homes vs community residential settings.* International journal of rehabilitation research. Internationale Zeitschrift fur Rehabilitationsforschung. Revue internationale de recherches de readaptation, 2004. **27**(4): p. 289-96.
15. Eggleton, I., et al., *The impact of employment on the quality of life of people with an intellectual disability.* Journal of Vocational Rehabilitation, 1999. **13**(2): p. 95-107.
16. Kober, R. and I.R.C. Eggleton, *Factor stability of the Schalock and Keith (1993) Quality of Life Questionnaire.* Mental retardation, 2002. **40**(2): p. 157-65.
17. Kober, R. and I.R.C. Eggleton, *The effect of different types of employment on quality of life.* Journal of intellectual disability research : JIDR, 2005. **49**(Pt 10): p. 756-60.
18. Lachapelle, Y., et al., *The relationship between quality of life and self-determination: an international study.* Journal of intellectual disability research : JIDR, 2005. **49**(Pt 10): p. 740-4.
19. Garcia-Villamisar, D., J. Dattilo, and J.L. Matson, *Quality of life as a mediator between behavioral challenges and autistic traits for adults with intellectual disabilities.* Research in Autism Spectrum Disorders, 2013. **7**(5): p. 624-629.
20. García-Villamisar, D.A. and J. Dattilo *Effects of a leisure programme on quality of life and stress of individuals with ASD*. Journal of intellectual disability research, 2010. **54**, 611-619 DOI: 10.1111/j.1365-2788.2010.01289.x.
21. Rapley, M. and L. Hopgood, *Quality of life in a community-based service in rural Australia.* Journal of Intellectual and Developmental Disability, 1997. **22**(2): p. 125-141.
22. van Nieuwpoort, I.C., et al., *The relationship between IGF-I concentration, cognitive function and quality of life in adults with Prader-Willi syndrome.* Hormones and behavior, 2011. **59**(4): p. 444-50.
23. Bergström, H., et al. *A multi-component universal intervention to improve diet and physical activity among adults with intellectual disabilities in community residences: a*

cluster randomised controlled trial. Research in developmental disabilities, 2013. **34**, 3847-3857 DOI: 10.1016/j.ridd.2013.07.019.

24. Bergstrom, H., et al., *Psychometric evaluation of a scale to assess satisfaction with life among people with intellectual disabilities living in community residences.* Journal of intellectual disability research : JIDR, 2013. **57**(3): p. 250-6.
25. Clark, L., et al., *Developing a Health-Related Quality-of-Life Measure for People With Intellectual Disability.* Intellectual and developmental disabilities, 2017. **55**(3): p. 140-153.
26. Roeden, J.M., M.A. Maaskant, and L.M. Curfs *Processes and effects of solution-focused brief therapy in people with intellectual disabilities: a controlled study*. Journal of intellectual disability research, 2014. **58**, 307-320 DOI: 10.1111/jir.12038.
27. Scheifes, A., et al., *Adverse events and the relation with quality of life in adults with intellectual disability and challenging behaviour using psychotropic drugs.* Research in developmental disabilities, 2016. **49-50**: p. 13-21.
28. Jeffery, C. and B. Hurtado, *EVALUATING SERVICE USERS' QUALITY OF LIFE.* Learning Disability Practice, 2015. **18**(2): p. 16-21.
29. Bai, X., et al. *Effectiveness of a life story work program on older adults with intellectual disabilities*. Clinical interventions in aging, 2014. **9**, 1865-1872 DOI: 10.2147/CIA.S56617.
30. García-Villamisar, D., J. Dattilo, and C. Muela *Effects of therapeutic recreation on adults with ASD and ID: a preliminary randomized control trial*. Journal of intellectual disability research, 2017. **61**, 325-340 DOI: 10.1111/jir.12320.
31. Garcia-Villamisar, D., J. Dattilo, and C. Muela *Effects of B-Active2 on Balance, Gait, Stress, and Well-Being of Adults With Autism Spectrum Disorders and Intellectual Disability: a Controlled Trial*. Adapted physical activity quarterly, 2017. **34**, 125-140 DOI: 10.1123/apaq.2015-0071.
32. Gur, A., *Challenging behavior, functioning difficulties, and quality of life of adults with intellectual disabilities.* International Journal of Developmental Disabilities, 2018. **64**(1): p. 45-52.
33. McGillivray, J.A., et al., *The utility of the Personal Wellbeing Index Intellectual Disability scale in an Australian sample.* Journal of Applied Research in Intellectual Disabilities, 2009. **22**(3): p. 276-286.
34. Balboni, G., et al., *The assessment of the quality of life of adults with intellectual disability: the use of self-report and report of others assessment strategies.* Research in developmental disabilities, 2013. **34**(11): p. 4248-54.

35. Claes, C., et al., *The influence of supports strategies, environmental factors, and client characteristics on quality of life-related personal outcomes.* Research in developmental disabilities, 2012. **33**(1): p. 96-103.

36. Guardia-Olmos, J., et al., *Item response theory analysis applied to the Spanish version of the Personal Outcomes Scale.* Journal of intellectual disability research : JIDR, 2017. **61**(11): p. 1021-1033.

37. Lombardi, M., et al., *Factors predicting quality of life for people with intellectual disability: Results from the ANFFAS study in Italy.* Journal of Intellectual & Developmental Disability, 2016. **41**(4): p. 338-347.

38. Memisevic, H., et al., *Quality of life of people with disabilities in Bosnia and Herzegovina-is there a moderating effect of the health status?* Scandinavian Journal of Disability Research, 2017. **19**(4): p. 375-382.

39. Simoes, C. and S. Santos, *Comparing the quality of life of adults with and without intellectual disability.* Journal of Intellectual Disability Research, 2016. **60**(4): p. 378-388.

40. Simões, C. and S. Santos, *The quality of life perceptions of people with intellectual disability and their proxies.* Journal of Intellectual & Developmental Disability, 2016. **41**(4): p. 311-323.

41. Simoes, C., S. Santos, and C. Claes, *Quality of life assessment in intellectual disabilities: the Escala Pessoal de Resultados versus the World Health Quality of Life-BREF.* Research in developmental disabilities, 2015. **37**: p. 171-81.
