# Supplementary material for: Adapting the EQ-5D-3L for adults with mild to moderate learning disabilities
Source: Health Qual Life Outcomes. 2024 Apr 29;22:37. doi: 10.1186/s12955-024-02254-x (PMC11059748; doi:10.1186/s12955-024-02254-x)
Supplement: Supplementary file 2 — Supplementary Material 2. [file 12955_2024_2254_MOESM2_ESM.pdf]

Appendix 2:

Adapting EQ-5D for adults with a learning disability

Semi-structured interview

In this first part of the interview, I'd like to ask you for some information about yourself and your experience with people with learning disabilities.
I would like to know about the characteristics of the people who complete this interview to check that we have a representative sample.
I would also like to check if people with different experiences have different opinions.
All data will be anonymised.

Think Aloud task begins:
A "warm-up exercise" to practice the think-aloud method will be conducted. This first task the participant will be asked to think-aloud and consider their activities that day up to the point of the meeting.
The interview will then proceed to presenting the EQ-5D to the participant. First the EQ-5D-3L and 5L will be presented in their original form for completion by the participant. They will be asked to imagine completing these measures from the perspective of the person they support. Should the participant have difficulty reading or problems with vision, the interviewer will read each sentence aloud. The researcher will remind the participant that they need to speak aloud as they complete the measure. They will be advised that paraphrasing (rephrasing/rewording) is acceptable. The interviewer will refrain from interrupting or distracting the participant, however, should the participant fall silent for ten seconds or more the interviewer will prompt them to keep speaking. Some participants are expected to experience difficulty at this stage and will be guided and prompted as the interviewer deems suitable. The participant will be informed that they can criticise the measure should they wish to, as the aim of the research is to find out what problems are faced by someone who has a learning disability when completing the measure.
After the participant has completed this stage the interview will proceed to a short discussion phase, taking the form of a semi-structured interview.

- The interviewer will ask probing questions about specific areas of the EQ-5D the participant may have had difficulty with.

- The participant will be asked about the wording of each domain and what their experience of the domain is on a day to day basis.

- Discussion will include what the participant thought of the questions, including which questions they found off-putting or confusing.

- The participants will be asked if they think there is anything missing from the measure which might influence the quality of life or “having a good life” for the person with learning disabilities that they support.

End of interview: Any further thoughts. Anything they feel they’d like to clarify?

Q. Would it be OK to contact you about the focus group?

End of survey

Thank you for your participation in this interview.

Your responses will contribute to the results and support the development of a version of the EQ-5D for people with learning disabilities.

INFORMED CONSENT STATEMENT

You are being invited to participate in a research study titled “Estimating health-related quality of life for adults with learning disabilities”. This research is being carried out by a researcher, John O’Dwyer, from the University of Leeds.

Thank you for your participation in this research.

In this interview you will be presented with a quality of life questionnaire which requires you to reflect on your experiences of health including pain and illness, which for some people may raise topics considered to be sensitive, embarrassing or otherwise upsetting. Previous experience suggests that although the topic may be distressing, participants find it a positive experience to express their views in a confidential environment.

Your participation in this study is entirely voluntary and you can withdraw at any time, however please be advised that any data you have provided up to that point will be kept. All data will be anonymised. Please be advised that due to the anonymous nature of this survey we will be unable to return any data to you if requested to do so.

We believe there are no known risks associated with this research study; however, as with any research related activity the risk of a breach is always possible. To the best of our ability your participation in this study will remain confidential, and only anonymised data will be published.

We will minimise any risks by holding electronic data on a secure server at the University of Leeds and will comply with all aspects of the 1998 Data Protection Act with data destroyed after 10 years.

There are no right or wrong answers, we are just interested in what you think.

Participant questionnaire

Age

1. How old are you? …………….years

Sex

1. Are you (*please tick*)

Male ☐

Female ☐

Social Support

2. Please tick the box that best applies to your marital status (*please tick*)

Married ☐	Widowed ☐
Cohabiting ☐	Single ☐
Separated/Divorced ☐	

Employment

3. Please, describe your current employment status (*please tick*)

Working full time (more than 30 hours per week) ☐	At home and not looking for paid employment (e.g. looking after your home, family or other dependants) ☐
Working part time (less than 30 hours per week) ☐	Unemployed and looking for work ☐
Unable to work due to illness / disability ☐	Student ☐
Retired ☐	Other ☐

Education

4. Did your education continue after the minimum school leaving age? (*please tick*)

☐ Yes ☐ No

5. Do you have a degree or equivalent professional qualification? (*please tick*)

☐ Yes ☐ No

Information on the person you provide support for

6. How many years have you been a carer/supporter?

…………..Months …………Years

7. What is your relationship to the person you provide support for? (*please tick*)

I am their parent or guardian	☐	I am their neighbour or friend	☐
I am their brother or sister	☐	I work in social care services	☐
I am their child	☐	Other	☐

If your relationship is "other" please provide details:

…………………………………………………………………………………………………..
