# Supplementary material for: Adapting the EQ-5D-3L for adults with mild to moderate learning disabilities
Source: Health Qual Life Outcomes. 2024 Apr 29;22:37. doi: 10.1186/s12955-024-02254-x (PMC11059748; doi:10.1186/s12955-024-02254-x)
Supplement: Supplementary file 3 — Supplementary Material 3. [file 12955_2024_2254_MOESM3_ESM.pdf]

Appendix 3:

UNIVERSITY OF LEEDS

Leeds QoL

Exploring the way quality of life is measured for adults with a learning disability

Information Sheet:

Think Aloud Session & Focus Group

Carers/Supporters

Hello!

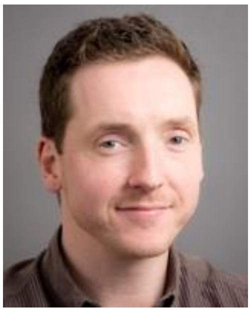

My name is John O'Dwyer.

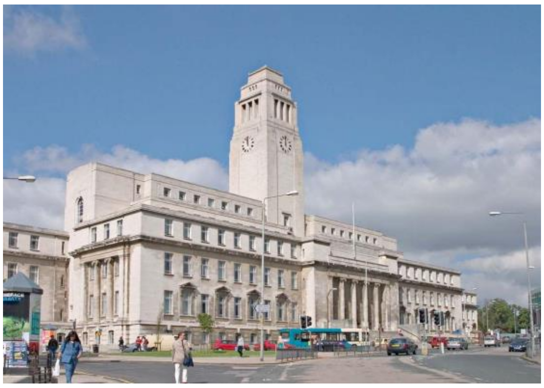

I am a researcher at the University of Leeds.

I would like your help with my research.

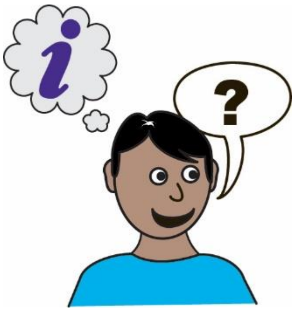

This short book tells you more about the research.

What is research?

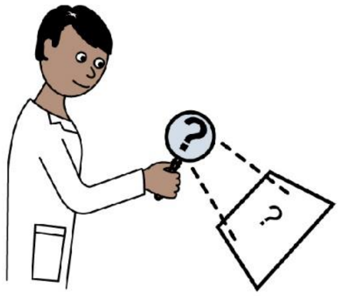

Research is when people try to find out about how something works or how it could work better.

Researchers are people who do research as their job.

Why am I doing research?

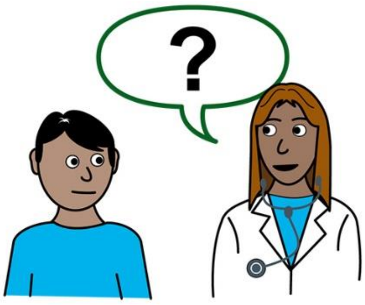

Doctors, nurses and other people working in health services want people with a learning disability to get the right care.

What is this research about?

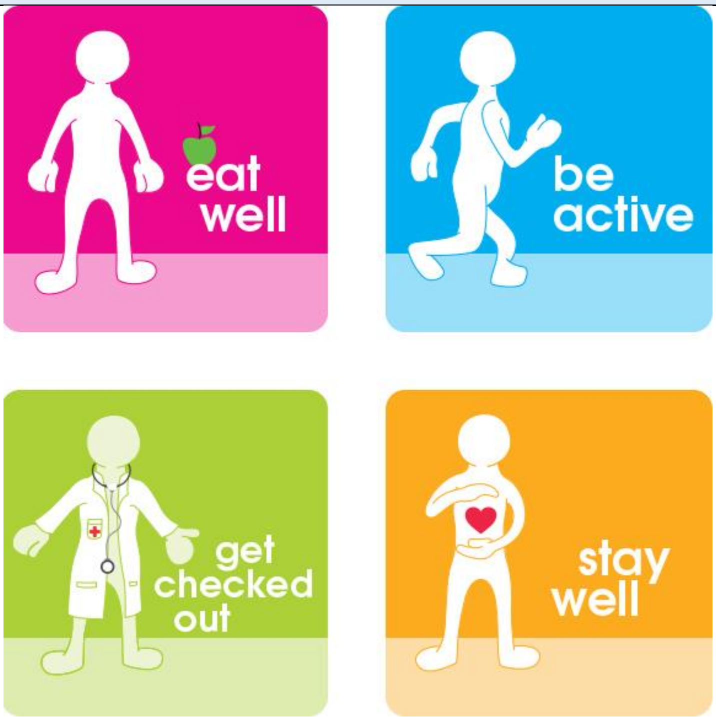

This research is about how to help people answer questions about how happy or healthy they are and how good a life they are having.

This is called “quality of life”.

I want to find a way that everyone can fill in a form about their quality of life.

Sometimes people have difficulty completing forms.

What do I want to know?	
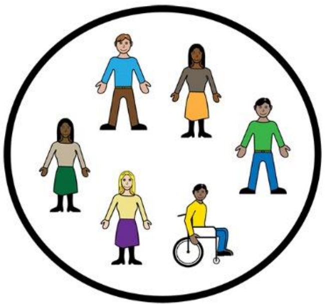	I need to know if people can complete a form about quality of life.
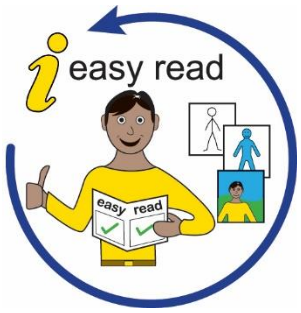	I want to know what words to use to make the form easier so everyone can answer it.
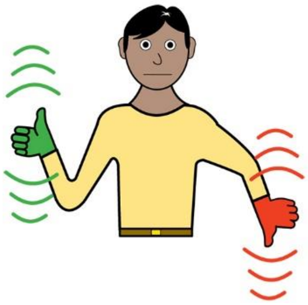	I want to know if the questions that are asked are the right questions.<br><br>Or is there anything you might add or take out?

Do you have to take part?

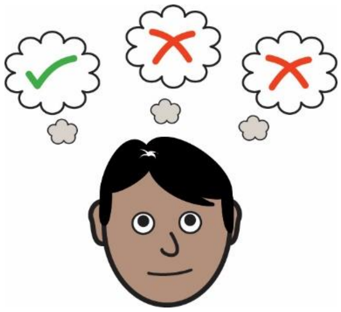

You can choose 'Yes' or 'No'.

It is up to you if you want to take part in the research, or not.

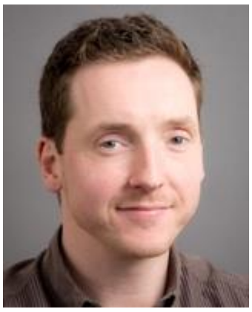

John

I will contact you and ask you if you want to take part.

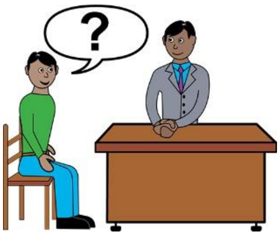

Before you choose you can ask any questions you want.

Help Deciding

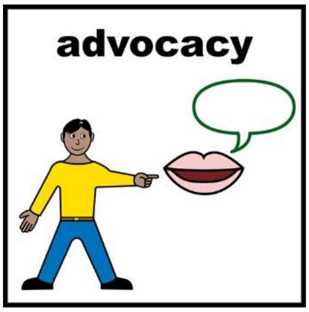

An advocate is someone who will speak on your behalf if you want them to.

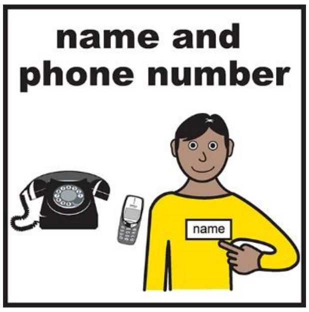

You will be given the name and phone number of someone from an advocacy group who can talk about the research with you.

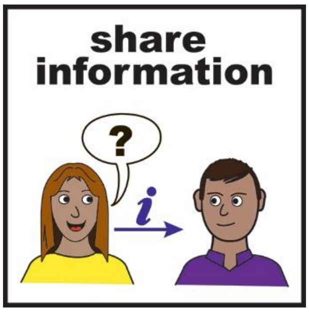

They can find out information for you and support you with making choices about being part of this research.

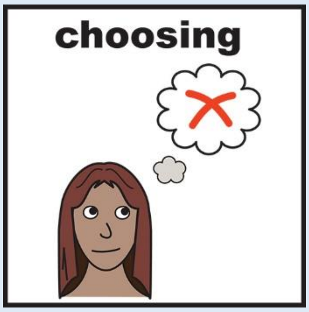 	**If you choose “No”**
**Choosing No is OK**	You can decide no.<br><br>If you think “No, I don’t want to take part” that’s ok. You don’t need to do anything else.<br>Just tell me “no” when I contact you.

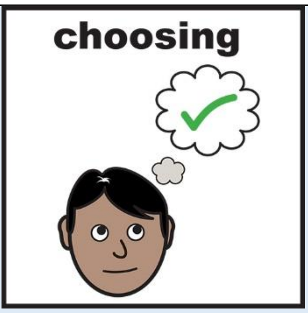

If you choose “Yes”

I will contact you using the contact details you provide.

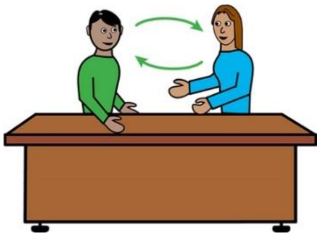

I will arrange to meet you at a place where you feel OK, at home or at a place that you like. You can choose where to meet me.

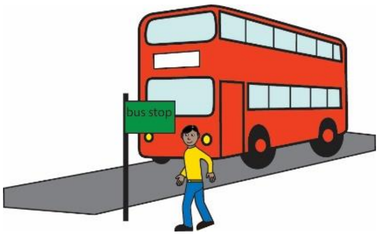

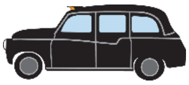

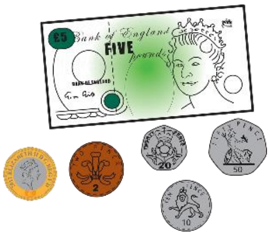

I can pay travel costs, like bus, train or taxi fares. I will not be able to pay you for taking part.

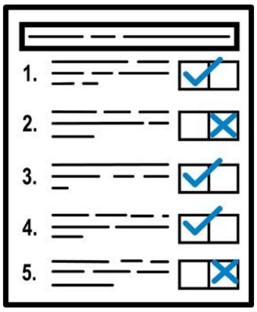

I will ask you to sign a form to say you are happy to take part. A copy of this form will be kept by me, no one else will see it.

I will send you a copy of the form.

The research team

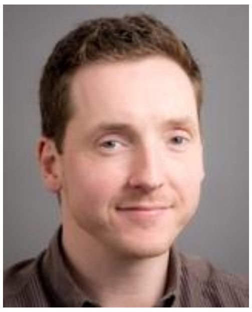

John

My name is John O’Dwyer. I am a researcher at the University of Leeds.

Claire Hulme, Louise Bryant, David Meads and Paul Kind are working with me on this research.

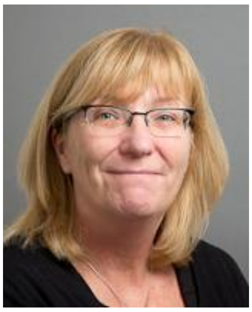

Claire

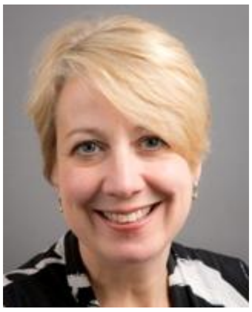

Louise

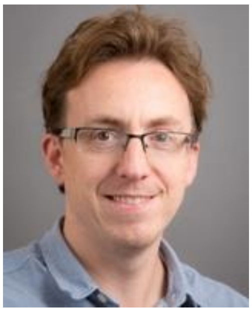

David

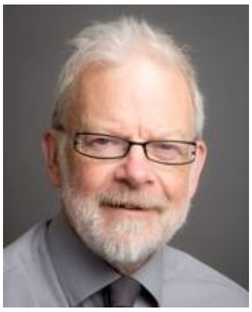

Paul

What happens first?

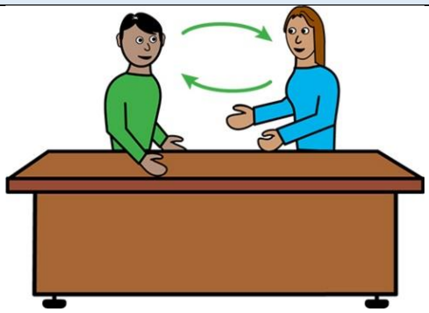

I will meet with you. I will ask you some questions about the research. I will explain what happens next.

At any time, if you don't want to help any more you can tell me.

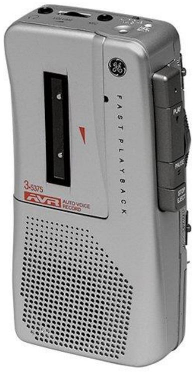

I will record what people say using a tape recorder.

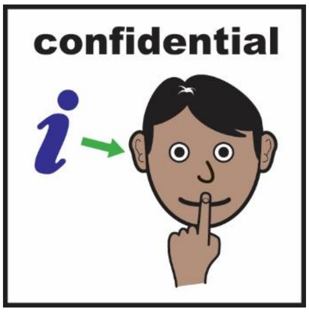

Nobody outside the research team will hear the recording.

The recording will be deleted once the research finishes.

What happens next?

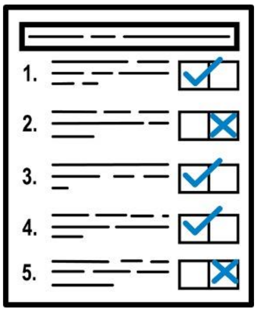

If you say OK to answering the questions then you will get a form to complete.

I will ask you to talk about the form aloud and what you think about it.

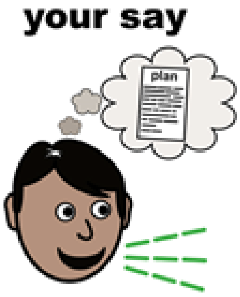

I can help you to read the form if you like. I can explain what each question asks.

What happens next?

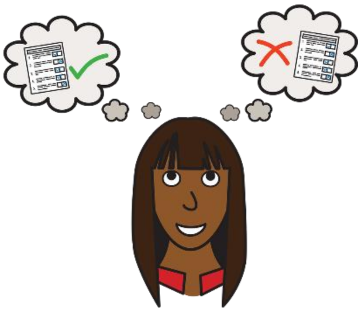

I will ask you to think about what matters to someone with a learning disability in terms of their quality of life.

I will ask you if there is anything to be added or taken out of the form.

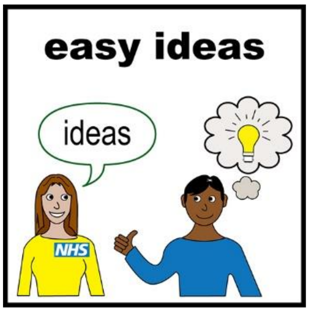

I will ask you about what you think might make the form easier for someone with a learning disability to complete.

There are no right or wrong answers. I just want to know what you think.

What happens then? – the Focus Group

A few weeks later you will be invited to come and meet with other carers and supporters of adults with learning disabilities.

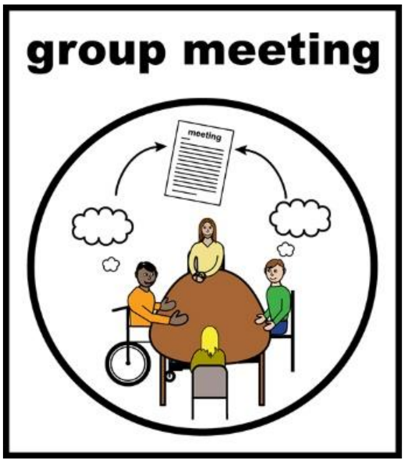

I will ask you and the other supporters to talk about the form and what matters to someone with a learning disability in terms of their quality of life.

You will see and hear what other carers and supporters thought about the form.

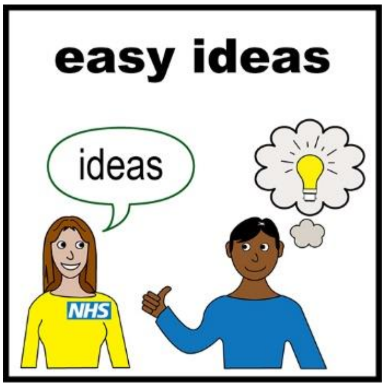

You will not have to come to this meeting if you do not want to.

There are no right or wrong answers. I just want to know what you think.

What if you want more information?

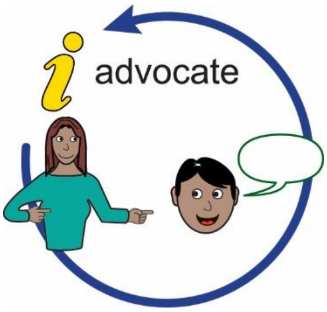

You can talk with an advocate at any time to help answer any questions.

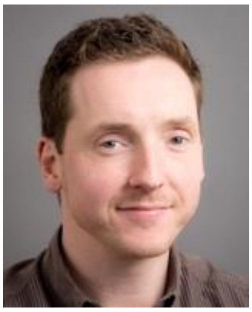

John

You can also ask me for more information.

What happens after the research finishes?

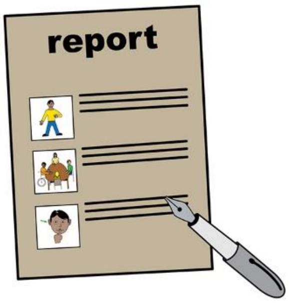

After more people have told me what they think of the form I will write about what I have learnt.

I will tell people what I have found out about the form.

Keeping things private

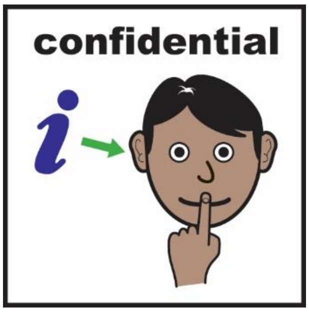

I will write down some things that you tell me. If I show this to other people it will not have your name on it.

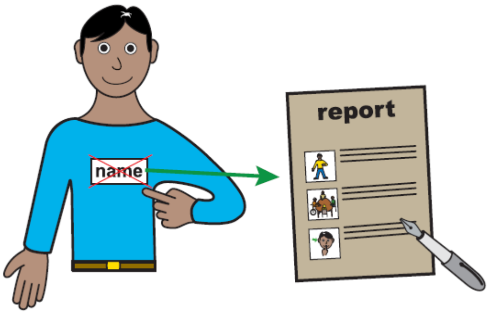

I will not tell other people your name.

Your name will not be in the report. Only the researcher will know what you said.

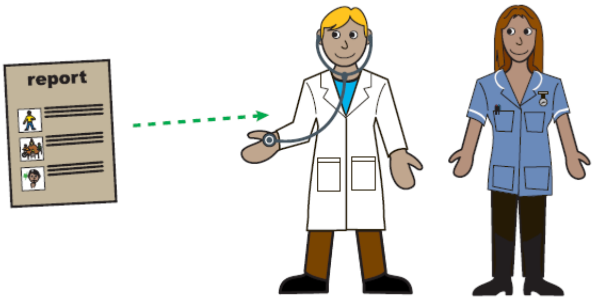

The report will be for other researchers, doctors, nurses, people who work with people with a learning disability and for the people who paid for the research.

Changing your mind

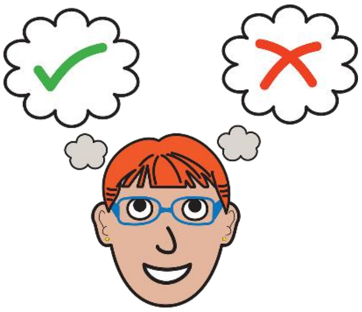

Even if you say yes to taking part in this research, you can change your mind at any time.

You have the right to stop being in the research at any point.

The care of the person you support will stay the same as it is now.

What might be some good things about taking part?

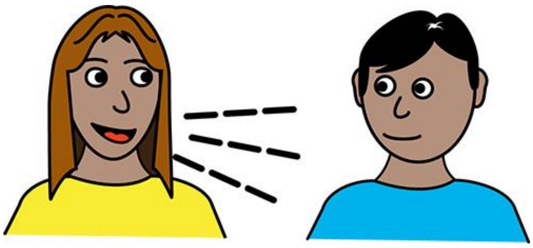

Some people like taking part in research.

Some people like talking about things and find it helps them.

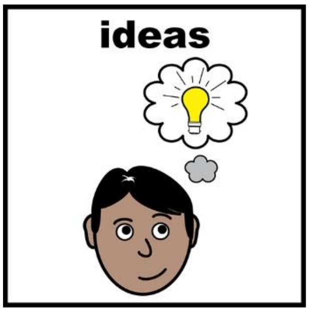

You may be helping people with a learning disability by sharing your ideas with us.

What might be some bad things about taking part?

Some people find talking about things a bit upsetting. If you are upset you can let the researcher know.

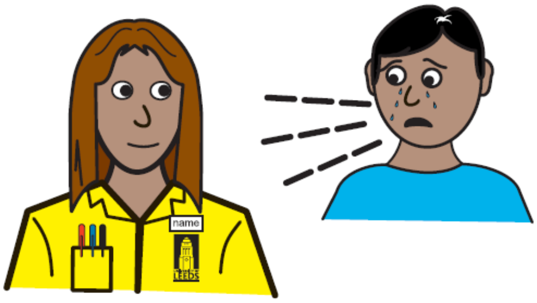

Some people don't like talking about what makes the person they support unhealthy or unhappy. If you don't like talking about these things please tell the researcher.

Helping us with this research will take up some of your time so make sure you are ok with this.

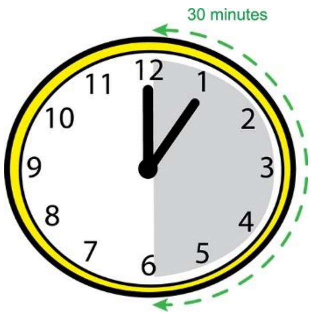

It should take about 30 minutes for the 1-to-1 meeting, and one hour for the group meeting, if you choose to take part.

Who is paying for this research?

This research is being paid for by The National Institute for Health Research (NIHR).

NIHR supports people like me to do research across the NHS.

The government gives them money and they have then given money to the University of Leeds to do the research.

Any questions?

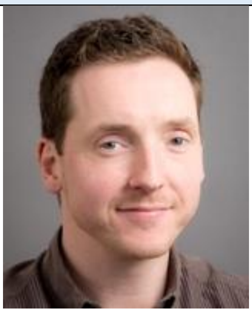

John

Any questions please contact John.

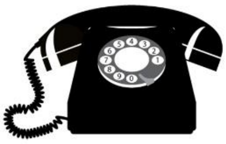

Telephone: 0113 343 6926

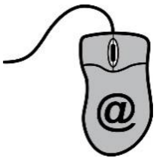

Email: j.odwyer@leeds.ac.uk

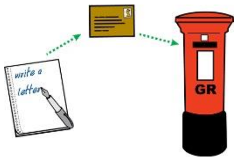

By post:

John O'Dwyer

Worsley Building

University of Leeds

Leeds

LS1 4JU

This space is for taking notes you want to make or questions you would like to ask
