# Supplementary material for: Adapting the EQ-5D-3L for adults with mild to moderate learning disabilities
Source: Health Qual Life Outcomes. 2024 Apr 29;22:37. doi: 10.1186/s12955-024-02254-x (PMC11059748; doi:10.1186/s12955-024-02254-x)
Supplement: Supplementary file 4 — Supplementary Material 4. [file 12955_2024_2254_MOESM4_ESM.pdf]

Which one describes you best?

<u>Walking about</u>

Thinking about **TODAY**

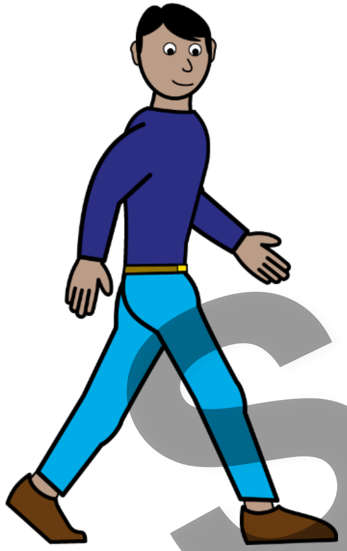

I can walk about on my own

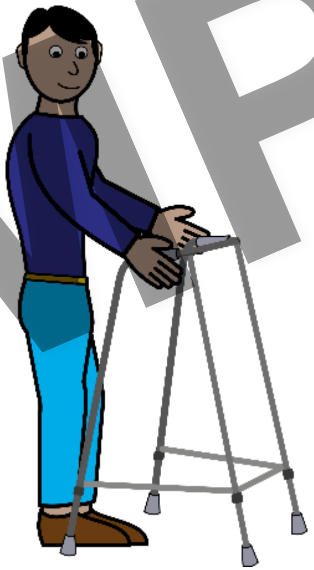

I need help to walk about

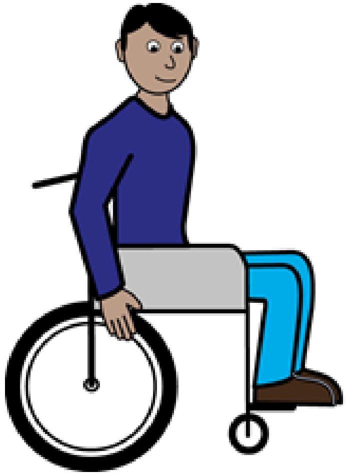

I cannot walk about

SAMPLE

© EuroQol Research Foundation. EQ-5D™ is a trade mark of the EuroQol Research Foundation.
This is a modified EQ-5D.

Looking after myself

Thinking about **TODAY**

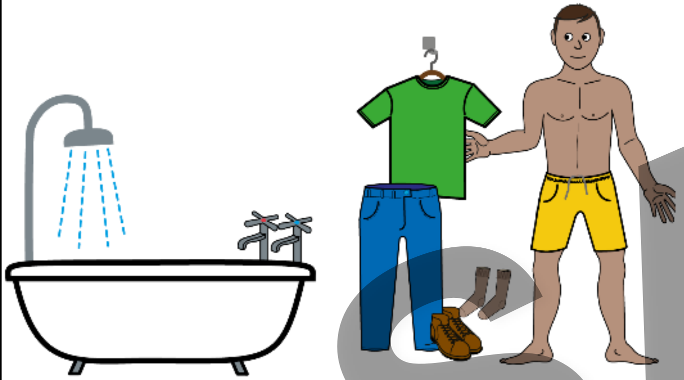

I can wash or dress myself on my own

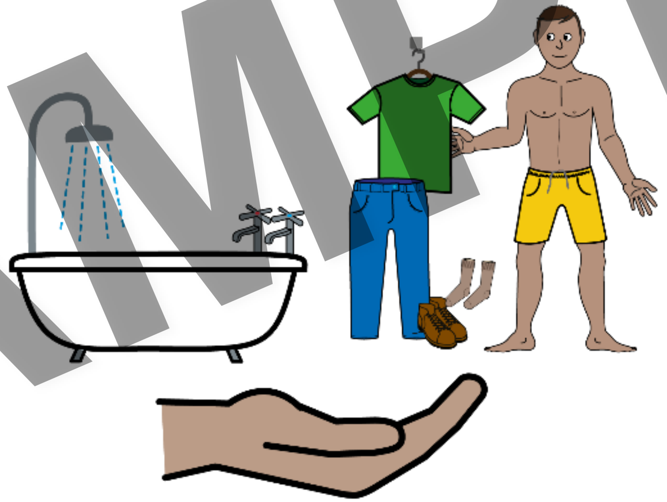

I need some help washing or dressing myself

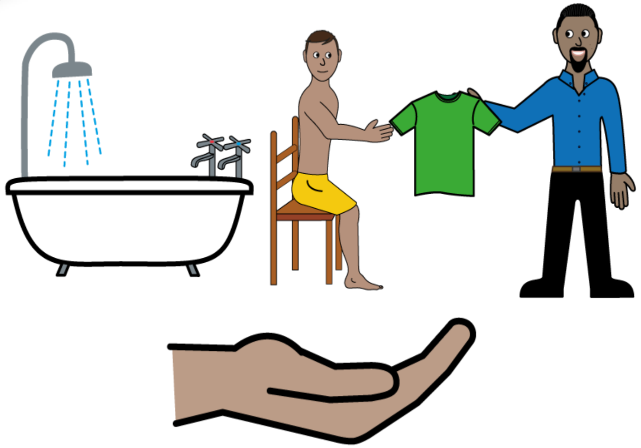

I need a lot of help from someone else to wash or dress myself

SAMPLE

© EuroQol Research Foundation. EQ-5D™ is a trade mark of the EuroQol Research Foundation.
This is a modified EQ-5D.

<u>Doing things I want to do</u>

Because of how I am **TODAY**…

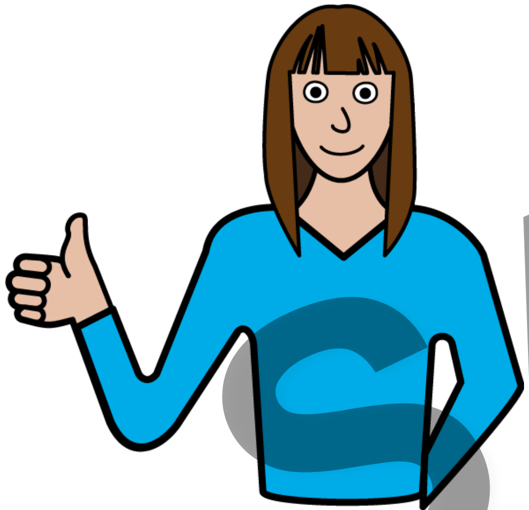

I can do the things I want to do

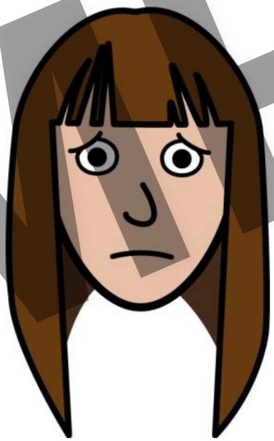

I have some difficulty doing the things I want to do

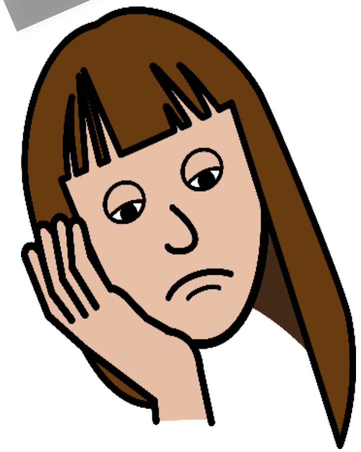

I cannot do the things I want to do

SAMPLE

© EuroQol Research Foundation. EQ-5D™ is a trade mark of the EuroQol Research Foundation.
This is a modified EQ-5D.

<u>Pain</u>

Thinking about **TODAY**

SAMPLE

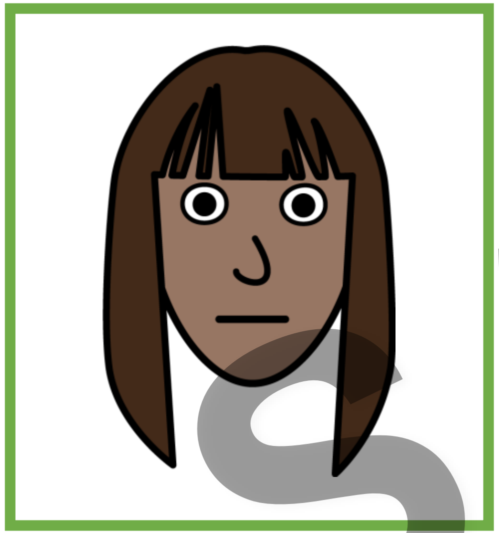

I have no pain

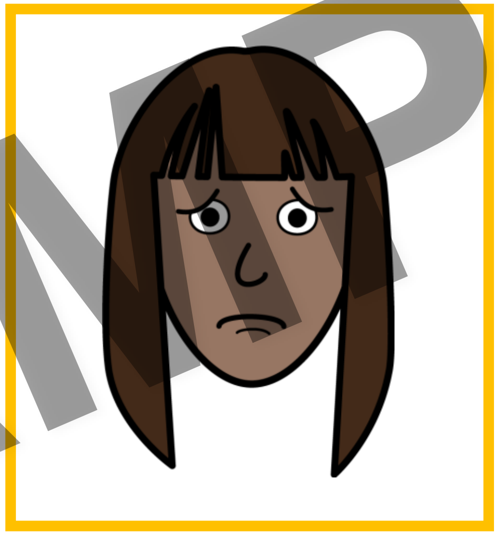

I have some pain

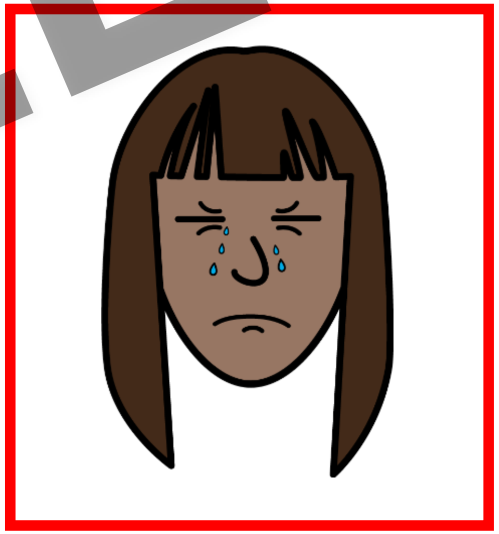

I have a lot of pain

© EuroQol Research Foundation. EQ-5D™ is a trade mark of the EuroQol Research Foundation.
This is a modified EQ-5D.

SAMPLE

Feeling worried, sad or unhappy

Thinking about **TODAY**

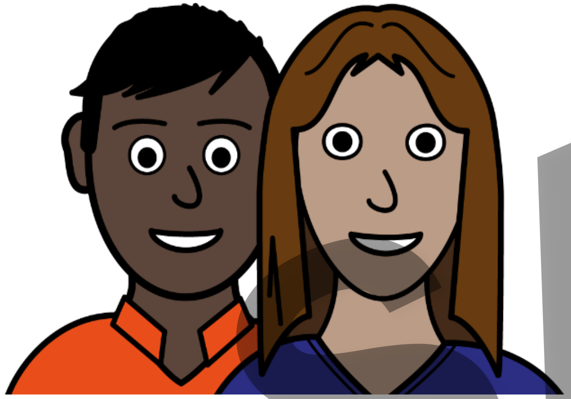

I am feeling OK

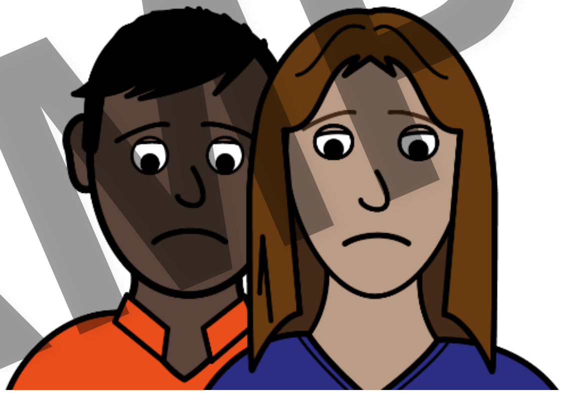

I am a bit worried, sad or unhappy

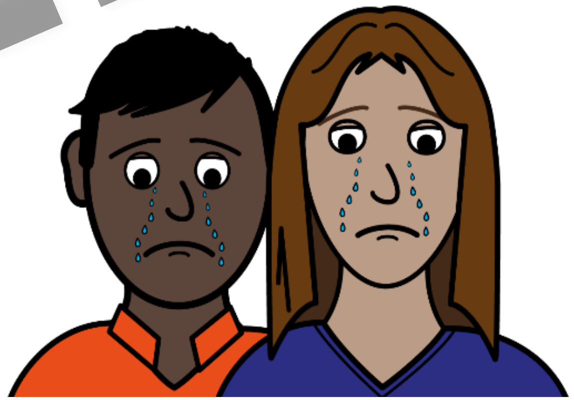

I am very worried, sad or unhappy

© EuroQol Research Foundation. EQ-5D™ is a trade mark of the EuroQol Research Foundation.
This is a modified EQ-5D.

We would like to know how you are **TODAY.**

If **10** is the BEST you could be and

0 is the **WORST (bad)** you could be,

which number shows how you are **TODAY**?

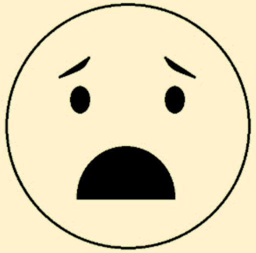

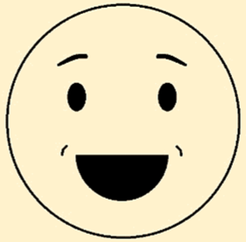

0	1	2	3	4	5	6	7	8	9	10
**WORST**										**BEST**

SAMPLE

© EuroQol Research Foundation. EQ-5D™ is a trade mark of the EuroQol Research Foundation.
This is a modified EQ-5D.
